# A 5-year Survey Reveals Increased Susceptibility to Glycopeptides for Methicillin-Resistant *Staphylococcus aureus* Isolates from *Staphylococcus aureus* Bacteremia Patients in a Chinese Burn Center

**DOI:** 10.3389/fmicb.2017.02531

**Published:** 2017-12-14

**Authors:** Bei Jiang, Supeng Yin, Bo You, Guangtao Huang, Zichen Yang, Yulong Zhang, Yu Chen, Jing Chen, Zhiqiang Yuan, Xiancai Rao, Xiaomei Hu, Yali Gong, Yizhi Peng

**Affiliations:** ^1^State Key Laboratory of Trauma, Burns and Combined Injury, Institute of Burn Research, Southwest Hospital, Third Military Medical University, Chongqing, China; ^2^Department of Microbiology, College of Basic Medical Sciences, Third Military Medical University, Chongqing, China

**Keywords:** methicillin-resistant *Staphylococcus aureus* (MRSA), burn, molecular epidemiology, glycopeptides, daptomycin, MIC creep

## Abstract

Methicillin-resistant *Staphylococcus aureus* (MRSA) infections are prevalent in burn wards, and are especially serious in *S. aureus* bacteremia (SAB) patients. Glycopeptides and daptomycin are effective against MRSA infections, but MIC creeps can reduce their efficacy. Our object was to perform a molecular epidemiological investigation of *S. aureus* isolates in our burn center and to evaluate MICs for antimicrobials against SAB-associated MRSA isolates. A total of 259 *S. aureus* isolates, obtained from August 2011 to July 2016, were used in this study. Multiple molecular typing was used for molecular epidemiological analysis. E-tests were used to determine MICs of vancomycin, teicoplanin, and daptomycin for SAB-associated MRSA isolates. MIC values were stratified by collection date or source and compared. Spearman's test was used to analyze MICs correlations amongst tested antimicrobials. ST239-MRSA-III-t030-*agr*I clone was found to be dominant in both SAB and non-SAB patients, and significantly more in SAB patients (*P* < 0.0001). SAB-MRSA isolates exhibited decreased MICs for vancomycin, teicoplanin, and daptomycin during the 5-year period. Compared to those isolated from catheters or wounds, SAB-MRSA isolates from the bloodstream were less susceptible to vancomycin and daptomycin, but more susceptible to teicoplanin. MICs Correlation was found only between vancomycin and daptomycin in MRSA isolates from the bloodstream (rho = 0.250, *P* = 0.024). In conclusion, our results suggest that MRSA infections are still serious problems in burn centers. In contrast to most other studies, we observed increased susceptibility to glycopeptides and daptomycin against SAB-associated MRSA in our center from 2011 to 2016, suggesting the use of glycopeptides does not lead to MIC creeps. Isolates from different sites of the body may exhibit different levels of susceptibility and change trend over time for different antimicrobials, antimicrobials selection for MRSA infections should be considered comprehensively.

## Introduction

*Staphylococcus aureus* is an important human pathogen that causes infections at diverse sites in the body (Rasigade and Vandenesch, 2014), it is also the most frequently isolated Gram-positive bacterial species from burn patients (Song et al., 2001; Bayram et al., 2013). In recent years, *S. aureus* has attracted particular attention because of the prevalence of methicillin-resistant *S*. *aureus* (MRSA), which poses a serious public health risk (Cheng et al., 2013; Carrel et al., 2015). The concern is even greater in patients suffering burn-associated infections, since the isolation rate for MRSA in burn wards is much higher than in the general hospital population and the resistance to antimicrobials is more dangerous (Bahemia et al., 2015; Motallebi et al., 2016). Multiple molecular typing based on MRSA detection, staphylococcal cassette chromosome *mec* (*SCCmec*) typing, multilocus sequence typing (MLST), *spa* typing, and *agr* grouping was usually used in molecular epidemiological investigation for *S. aureus* (Cheng et al., 2013). In China, ST239-MRSA-III-t030-*agr*I is the most prevalent and persistent MRSA clone, and is a major challenge to clinical anti-infection therapy (Cheng et al., 2013; Li et al., 2013). Several factors contribute to its successful adaptation and survival. The clone often carries the portable *SCCmec* III, which can transfer drug resistance genes (Cheng et al., 2013). In addition, the ST239 clone may have adaptations that confer enhanced antimicrobial resistance (Cha et al., 2005; Chen et al., 2014), and ST239-MRSA is more likely to be classified as a heterogeneous vancomycin-intermediate *S. aureus* (hVISA) (Hu et al., 2015).

*S. aureus* bacteremia (SAB) is a common and severe infection (Naber, 2009). It is associated with high mortality and represents a major burden to both the patient and the health care system, especially when life-threatening complications such as infective endocarditis and metastatic infections occur (Naber, 2009). Due to susceptible factors such as loss of the skin barrier, immune deficiency, and the use of invasive catheters and monitoring devices, SAB in burn patients occurs more frequently, and is extremely serious in cases involving MRSA-associated SAB (Gang et al., 2000; Bahemia et al., 2015).

Vancomycin, a glycopeptide antimicrobial, has been in clinical use globally for over 50 years, and still serves as a first-line drug for treatment of SAB caused by MRSA (Choo and Chambers, 2016). However, many recent studies have documented an increase in the minimum inhibitory concentration (MIC) for vancomycin over time (“MIC creep”) (Steinkraus et al., 2007; Chang et al., 2015). Because vancomycin MIC creep is thought to be a potential contributor to increased treatment failure and mortality, new agents for effective therapy against MRSA are urgently needed (Chang et al., 2015; Choo and Chambers, 2016). In the USA, daptomycin, a lipopeptide class antimicrobial, is available as an effective alternative for invasive MRSA infection (Humphries et al., 2013). Daptomycin is seldom used in China primarily because its high cost is not reimbursed by medical insurance. Although the drug may have a promising market and widespread application in China, several studies have reported a correlation between vancomycin and daptomycin MICs, suggesting that decreased vancomycin susceptibility is linked to decreased susceptibility to daptomycin (Hsieh et al., 2016; San-Juan et al., 2016). If this is indeed the case, it will restrict the utility of daptomycin in China in the future. Teicoplanin is another glycopeptide antimicrobial used for serious infections caused by MRSA (Choo and Chambers, 2016). While having not been approved for use in the USA, it is used as commonly as vancomycin in Europe and many hospitals in China, and exhibits an efficacy comparable to vancomycin (Chang et al., 2012; Choo and Chambers, 2016). Decreased teicoplanin susceptibility has also been suggested as a contributor to treatment failure of MRSA infections (Chang et al., 2012). Because MIC creeps may affect the prognosis of MRSA-associated SAB (Chang et al., 2012, 2015), for clinical MRSA isolates, the evaluation of MICs for glycopeptides and daptomycin over time is a high priority.

In this study, we first analyzed the molecular epidemiological characteristics of clinical *S. aureus* isolates from SAB and non-SAB patients in our center. Since our result suggested that SAB patients presented significantly more ST239-MRSA-III-t030-*agr*I infections, we determined MICs for all the MRSA isolates from SAB patients using E-tests, and evaluated antimicrobial MICs of these isolates according to their collection dates and sources. The tested antimicrobials included vancomycin and teicoplanin, the two most commonly used glycopeptides in our center. Daptomycin, which is never used in our hospital, was also tested to analyze the correlation of MICs amongst the three antimicrobials.

## Patients and methods

### Ethics statement

The study was approved by the Committee of the First Affiliated Hospital of Third Military Medical University, China. No written informed consent was required because all the patients were anonymous and any other personal information was not used in this study.

### Study design and bacterial isolates

This study was conducted in the 150-bed burn center of Southwest Hospital in China. From August 2011 to July 2016, 259 clinical isolates derived from SAB and non-SAB patients were enrolled for analysis. Strains from SAB patients were isolated from the bloodstream (group BB), catheters (group CB), or wounds (group WB), the three sites where *S. aureus* was most commonly isolated from SAB patients in our center. All patients in groups CB and WB were included in group BB. Strains from non-SAB patients were isolated from wounds (group WN), the most common infection site in burn patients. The numbers of isolates in each group and collection dates are summarized in Table 1. All isolates were identified as *S. aureus* by phenotypic methods (API staphy system, Biomerieux) and confirmed by PCR amplification of the *femB* gene. SAB was defined as an episode of fever with at least one peripherally-drawn blood culture that was positive for *S. aureus* (Ponce-De-Leon et al., 2010). Only the first positive culture in the course of infection was enrolled for analysis.

**Table 1:** Distribution of isolates based on source and sampling time.

	**2011.08–2012.07**	**2012.08–2013.07**	**2013.08–2014.07**	**2014.08–2015.07**	**2015.08–2016.08**	**Total**
BB	19	10	30	18	10	87
CB	3	3	7	6	7	26
WB	12	7	17	12	8	56
WN	7	19	24	23	17	90
Total	41	39	78	59	42	259

*BB, isolates from bloodstream of bacteremia patients; CB, isolates from catheter of bacteremia patients; WB, isolates from wound of bacteremia patients; WN, isolates from wound of non-bacteremia patients*.

### Molecular typing

MRSA was identified by duplex PCR using *femB* and *mecA* as reference genes (Kobayashi et al., 1994). *SCCmec* typing of MRSA was performed using four unique and specific pairs of primers as described previously (Kondo et al., 2007). For MLST, ST239 strains were first screened using two sets of primers designed by Feil et al. (2008). Other sequence types (STs) were determined by PCR amplification of seven housekeeping genes (*arcC, aroE, glpF, gmk, pta, tpi*, and *yqil*), the reaction products were sequenced and aligned to sequences available at the Multi Locus Sequence Typing website (http://saureus.mlst.net/) (Cheng et al., 2013). The *spa* type was identified by amplifying and sequencing the polymorphic X-region of the *spa* gene (Harmsen et al., 2003). The *agr* group was identified by PCR using one common and four specific primers, followed by electrophoresis, as previously described (Bardiau et al., 2016).

### Antimicrobial susceptibility tests

All MRSA isolates from SAB patients were tested for susceptibility to vancomycin, teicoplanin, and daptomycin by standard E-test according to the manufacturer's guidelines (bioMérieux, France). All E-test results were visually read by two independent observers, who were blinded to each other and to the grouping situations. Interpretive standards for antimicrobial susceptibility followed criteria established by the Clinical and Laboratory Standards Institute (CLSI). *S. aureus* ATCC 29213 was used as the control strain.

### Statistical analysis

IBM SPSS Statistics 19.0 (IBM, Chicago, IL, USA) and GraphPad Prism 6.0 software (San Diego, CA, USA) were used for statistical analysis. We used Student's *t*-test to compare continuous variables, and χ^2^ test to compare categorical variations. Correlations between E-test MICs were calculated using Spearman's rho (ρ) test. All statistical tests were two-sided and the threshold for statistical significance was *P* < 0.05.

## Results

### Molecular epidemiological characteristics of *S. aureus* isolates in the burn center

A total of 239 MRSA isolates (239/259, 92.28%) were identified from patients in our burn center. As shown in Table 2, MRSA was significantly more frequently isolated from SAB patients than from non-SAB patients (95.86 vs. 85.56%, respectively; *P* = 0.0031). Overwhelming majority of MRSA isolates were classified as *SCCmec* III (230/239, 96.23%). ST239 was the predominant MLST type (238/259, 91.89%), and was more likely to be isolated from SAB patients (95.86 vs. 84.44%, respectively; *P* = 0.0014). The *spa* type t030 (93.49 vs. 77.78%, respectively; *P* = 0.0002) and *agr* group I (99.41 vs. 94.44%, respectively; *P* = 0.0362) were also significantly more abundant in SAB patients. For isolates from wounds, the most common isolation site for *S. aureus* in burn wards, SAB patients were more likely to be infected by the ST239-MRSA-t030 clone compared to non-SAB patients. The ST239-MRSA-III-t030-*agr*I clone, the most prevalent MRSA clone in China and closely related to antimicrobial resistance (Cheng et al., 2013; Li et al., 2013), was significantly more frequent in SAB patients (92.90 vs. 70.00%, respectively; *P* < 0.0001).

**Table 2:** Molecular epidemiological characteristics of enrolled isolates.

	**BB (PROP, PCT, *P*^a^)**	**CB (PROP, PCT, *P*^a^)**	**WB (PROP, PCT, *P*^a^)**	**TB (PROP, PCT, *P*^a^)**	**WN (PROP, PCT)**
MRSA	82/87, 94.25% *P* = 0.0556	26/26, 100% *P* = 0.0884	54/56, 96.43% ***P* = 0.0354**	162/169, 95.86% ***P* = 0.0031**	77/90, 85.56%
MRSA-*SCCmec* III	81/82, 98.78% *P* = 0.1843	25/26, 96.15% *P* = 0.9887	52/54, 96.30% *P* = 0.7609	158/162, 97.53% *P* = 0.2445	72/77, 93.51%
ST239	83/87, 95.40% ***P* = 0.0306**	25/26, 96.15% *P* = 0.2166	54/56, 96.43% ***P* = 0.0242**	162/169, 95.86% ***P* = 0.0014**	76/90, 84.44%
t030	82/87, 94.25% ***P* = 0.0017**	25/26, 96.15% *P* = 0.0637	51/56, 91.07% ***P* = 0.0381**	158/169, 93.49% ***P* = 0.0002**	70/90, 77.78%
*agr*-I	87/87, 100% *P* = 0.0757	26/26, 100% *P* = 0.4962	55/56, 98.21% *P* = 0.4920	168/169, 99.41% ***P* = 0.0362**	85/90, 94.44%
ST239-MRSA- III-t030-*agr* I	81/87, 93.1% ***P* < 0.0001**	25/26, 96.15% ***P* = 0.0060**	51/56, 91.07% ***P* = 0.0028**	157/169, 92.90% ***P* < 0.0001**	63/90, 70.00%

*TB, total isolates from SAB patients = BB + CB + WB; PROP, proportion; PCT, percentage*.

a *χ^2^- test with the data in group WN. Significant differences are in boldface*.

### Susceptibility of SAB-associated MRSA isolates to vancomycin, teicoplanin, and daptomycin, stratified by collection dates and sources

Because SAB is a dangerous infection in burn patients, and infections caused by clone ST239-MRSA-III-t030-*agr*I are significantly more frequent in SAB patients in our center, we sought to evaluate MICs for the three important antimicrobials against MRSA (vancomycin, teicoplanin, and daptomycin) amongst SAB-associated MRSA isolates. No resistant, intermediate-resistant or non-susceptible strain for any of the three antimicrobials was identified. When these MRSA isolates were stratified by collection dates, we were surprised to find that the susceptibility to vancomycin, teicoplanin, and daptomycin all tended to increase during the 5-year period (Table 3). Comparing to the data in the first 3 years, MIC_50_ values of vancomycin and teicoplanin, MIC_50_ and MIC_90_ values of daptomycin all decreased in the last 2 years. MIC_GM_ values and percentages of isolates with MIC > median values for the three antimicrobials all significantly decreased, especially for vancomycin (*P* = 0.0018 and *P* = 0.0002) and teicoplanin (*P* < 0.0001 and *P* < 0.0001), despite their frequent use for MRSA infections in our center.

**Table 3:** Susceptibility of SAB-MRSA to vancomycin, teicoplanin, and daptomycin, stratified by collection date.

		**1108–1207**	**1208–1307**	**1308–1407**	**1408–1507**	**1508–1607**	**1108–1407**	**1408–1607**	***P*-value**
		***n* = 33**	***n* = 20**	***n* = 54**	***n* = 32**	***n* = 23**	***n* = 107**	***n* = 55**	
VAN	MIC_50_ (mg/L)	1.5	1.0	1.0	1.0	1.0	1.5	1.0	–
	MIC_90_ (mg/L)	2.0	2.0	1.5	1.5	1.5	1.5	1.5	–
	MIC_GM_ (mg/L)	1.38	1.25	1.19	1.10	1.11	1.26	1.10	**0.0018^a^**
	MIC ≥ 1.5 (n, PCT)	27, 81.8%	10, 50.0%	28, 51.9%	11, 34.4%	5, 21.7%	64, 59.8%	16, 29.1%	**0.0002^b^**
TEC	MIC_50_ (mg/L)	1.0	0.5	1.0	0.5	0.5	1.0	0.5	-
	MIC_90_ (mg/L)	1.5	1.5	1.5	1.0	1.5	1.5	1.5	-
	MIC_GM_ (mg/L)	0.97	0.71	0.89	0.61	0.62	0.88	0.61	**<0.0001^a^**
	MIC ≥ 1 (n, PCT)	26, 78.8%	10, 50.0%	39, 72.2%	8, 25.0%	6, 26.1%	75, 70.1%	14, 25.5%	**<0.0001^b^**
DPC	MIC_50_ (mg/L)	0.38	0.5	0.5	0.38	0.5	0.5	0.38	–
	MIC_90_ (mg/L)	1.0	1.0	0.75	0.5	0.75	0.75	0.5	–
	MIC_GM_ (mg/L)	0.44	0.45	0.46	0.36	0.45	0.45	0.40	**0.0499^a^**
	MIC ≥ 0.5 (n, PCT)	16, 48.5%	11, 55.0%	30, 55.6%	8, 25.0%	12, 52.2%	57, 53.3%	20, 36.4%	**0.0413^b^**

a t-test;

b *χ^2^- test. MIC_GM_, geometric mean of MIC values; PCT, percentage*.

*Significant differences are in boldface*.

SAB-MRSA isolates from different sources presented different susceptibility profiles. As shown in Table 4, although no statistical difference was found, MRSA isolates from the bloodstream were less susceptible to vancomycin, reflected by higher values for MIC_50_, MIC_GM_, and the percentage of isolates with MIC > 1.5 mg/L (median value). In contrast, MRSA isolates from the bloodstream were more susceptible to teicoplanin. MIC_50_, MIC_90_, MIC_GM_, and the proportion of isolates with MIC > 1 mg/L (median value) were all lower in group BB. A *t*-test showed significantly lower teicoplanin MIC values in group BB compared to group CB (*P* = 0.0415) or WB (*P* = 0.0328). Susceptibility patterns of daptomycin and vancomycin were similar. Significant differences were observed between the MIC values (*P* = 0.0030) and the percentages of isolates with MIC > 0.5 mg/L (*P* = 0.0295) between group BB and WB.

**Table 4:** Susceptibility of SAB-MRSA to vancomycin, teicoplanin, and daptomycin, stratified by source.

		**BB (*n* = 82)**	**CB (*n* = 26)**	**WB (*n* = 54)**
VAN	MIC_50_ (mg/L)	1.5	1.0	1.0
	MIC_90_ (mg/L)	1.5	1.5	1.5
	MIC_GM_ (mg/L), *P*^a^	1.26, -	1.17, 0.1004	1.13, 0.1303
	MIC ≥ 1.5 (n, PTC, *P*^b^)	44, 53.7%, –	10, 38.5%, 0.2318	26, 48.2%, 0.5293
TEC	MIC_50_ (mg/L)	0.5	1.0	1.0
	MIC_90_ (mg/L)	1.5	2.0	1.5
	MIC_GM_ (mg/L), *P*^a^	0.72,–	0.88, **0.0415**	0.82, **0.0328**
	MIC ≥ 1 (n, PTC, *P*^b^)	39, 47.6%, –	17, 65.4%, 0.11	33, 61.1%, 0.12
DPC	MIC_50_ (mg/L)	0.5	0.38	0.38
	MIC_90_ (mg/L)	1.0	0.5	0.5
	MIC_GM_ (mg/L), *P*^a^	0.47, –	0.42, 0.0554	0.38, **0.0030**
	MIC ≥ 0.5 (n, PCT, *P*^b^)	46, 56.1%, –	11, 42.3%, 0.2197	20, 37.0%, **0.0295**

a t-test, group BB vs. group CB or WB;

b *χ^2^- test, group BB vs. group CB or WB*.

*No significant difference was found between group CB and WB*.

*MIC_GM_, geometric mean of MIC values; PCT, percentage. Significant differences are in boldface*.

### Correlation of MICs amongst vancomycin, teicoplanin, and daptomycin

Vancomycin MIC exhibited a trend of correlation with daptomycin MIC in SAB-MRSA isolates (Spearman's rho = 0.140, *P* = 0.076). Further analysis revealed that the two MICs were correlated significantly only for isolates from the bloodstream (Spearman's rho = 0.250, *P* = 0.024). In addition, correlations were not found between teicoplanin and daptomycin MICs, or between vancomycin and teicoplanin MICs (Table 5).

**Table 5:** Correlation analysis amongst vancomycin, teicoplanin, and daptomycin.

		**TB**	**BB**	**CB**	**WB**
VAN vs. DPC	rho	0.140	**0.250**	0.060	−0.023
	*P*	0.076	**0.024**	0.771	0.868
TEC vs. DPC	rho	0.033	0.051	0.153	0.070
	*P*	0.679	0.651	0.454	0.616
VAN vs. TEC	rho	0.090	0.143	0.284	0.006
	*P*	0.255	0.201	0.159	0.963

*TB, total isolates from SAB patients = BB + CB + WB. Analyzed by Spearman's rho (ρ) test. The rho and P values of the significant correlation are in boldface*.

## Discussion

Multiple molecular typing of *S. aureus* is an important tool in epidemiological studies and is useful for the monitoring and control of infection (Cheng et al., 2013). In this work, we first analyzed *S. aureus* isolates from SAB and non-SAB patients in our burn center over a 5-year period. We found that the overall prevalence rate of MRSA in our center was high (92.28%). This result was similar to burn survey reports by Song et al. (98%) from Korea (Song et al., 2001), Khosravi et al. (87.4%), and Parhizgari et al. (86.4%) from Iran (Khosravi et al., 2012; Parhizgari et al., 2016), but higher than that reported by Chen et al. (55.3%) from southeast China (Chen et al., 2012). In contrast to the molecular typing results of other common bacteria from burn patients, such as *Acinetobacter baumannii* and *Pseudomonas aeruginosa*, which often display diverse molecular types (Huang et al., 2016; de Almeida Silva et al., 2017), the molecular types of *S. aureus* in burn wards were typically simple according to previous studies (Chen et al., 2012; Parhizgari et al., 2016). In our center, almost all the MRSA strains carried *SCCmec* III (96.23%), which is a characteristic of nosocomial MRSA strains and is associated with the capacity to resist antimicrobials (Cheng et al., 2013). The dominant MLST type was ST239 [91.89%, (162+76)/259], suggesting its strong ability to adapt and persist, as mentioned previously (Table 2) (Cha et al., 2005; Chen et al., 2014). The circulation of limited number of *S. aureus* clones in burn wards may be due mainly to the compromised skin barrier, poor conditions of burn patients, and the strong adaptability of specific *S. aureus* clones. This underscores the importance of placing more emphasis on disinfection of burn wards and patients to eradicate or restrict circulating bacteria.

According to our results, SAB patients were significantly more likely to be infected by MRSA (*P* = 0.0031) and clone ST239-MRSA-III-t030-*agr*I (*P* < 0.0001) (Table 2). The latter is considered the most prevalent hospital-acquired MRSA clone in China, and is closely associated with antimicrobial resistance (Cheng et al., 2013; Li et al., 2013). Its presence places burn patients with SAB at greater risk, and makes it more difficult to cure SAB infections. Moreover, if MIC creeps occurs, the situation may get worse (Chang et al., 2015; Choo and Chambers, 2016). MIC creeps to glycopeptides and daptomycin have been widely reported. Chang et al. found that the percentage of isolates with a vancomycin MIC of 1 mg/L increased significantly from 2006 (37.0%) to 2010 (75.7%) in a Chinese hospital, and the high vancomycin MIC was associated with a higher failure rate of anti-infection therapy in these patients (Chang et al., 2015). Increased vancomycin MICs to MRSA were also reported by Steinkraus et al. (2007) and Wang et al. (2006) in their 5-year studies in the USA. Hsieh et al. also observed MIC creeps of MRSA to vancomycin, teicoplanin, and daptomycin in an 11-year period study (Hsieh et al., 2016). However, some studies did not find an upward MIC creep. Sancak et al. demonstrated that the distribution of MICs for vancomycin and daptomycin was stable in an 11-year period (Sancak et al., 2013). Reynolds et al. did not find an upward creep for vancomycin or teicoplanin in the UK and Ireland (Reynolds et al., 2012). To our best knowledge, decreasing MIC trends were rarely reported by now. Steinkraus et al. reported that daptomycin MICs to MRSA significantly decreased from 2001 to 2005 in a medical center (Steinkraus et al., 2007). Lu et al. found that vancomycin MICs among MRSA isolates in a Chinese tertiary hospital decreased in a 12-year period (Lu et al., 2016). However, drug usage information was not mentioned or could not be acquired in the two studies. In this study, vancomycin and teicoplanin were used in almost all the MRSA-SAB patients in our center, while daptomycin was never used. Our results showed that, all the three antimicrobials exhibited significant MICs decreases during the 5-year period, especially teicoplanin (*P* < 0.0001). These results suggest that long-term use of glycopeptides (vancomycin and teicoplanin) does not necessarily lead to upward MIC creeps for glycopeptides or daptomycin, which are consistent with Lu et al.'s (2016) and Steinkraus et al.'s (2007) studies, but contrast to most other studies. Infection types, enforcement of guidelines for the reasonable use of antimicrobials, time and dosage for antimicrobials administration may contribute to the MIC trends. More detailed and in-depth investigation is needed to explain why different MIC trends were observed in different studies, and this will provide greatly useful guidance for antimicrobials administration.

In burn patients with SAB, wound infection was considered as the major cause of invasive infection into the bloodstream (Pruitt et al., 1998), but previous studies did not evaluate the MICs of SAB isolates from different sites of the body. The present study showed that strains isolated from different sites in SAB patients present different susceptibility profiles to glycopeptides and daptomycin. In our results, MRSA isolates from the bloodstream exhibited decreased susceptibility to both vancomycin and daptomycin, compared to those isolated from catheters or wounds. Intriguingly, teicoplanin MICs showed an opposite trend. MIC values (*P* < 0.0001), and the number of isolates with MIC ≥ 1 mg/L (*P* < 0.0001), were both significantly lower in bloodstream isolates (Table 4). Our results suggest that teicoplanin may be a better choice for MRSA infections from the bloodstream. To efficiently defeat MRSA infections, clinicians should select drugs based on the susceptibility tests of bacteria isolated from different sites in the patient.

The correlations amongst MICs for vancomycin, teicoplanin, and daptomycin have been reported previously (Shoji et al., 2015; Hsieh et al., 2016; San-Juan et al., 2016). Hsieh et al. found that the vancomycin MIC was significantly correlated with teicoplanin, daptomycin, and teicoplanin MICs (Hsieh et al., 2016). Shoji et al. also observed that the vancomycin MIC was significantly correlated with MICs for both teicoplanin and daptomycin (Shoji et al., 2015). San-Juan et al. found a correlation between high MICs for vancomycin (>1.5 mg/L) and daptomycin (>0.5 mg/L) (San-Juan et al., 2016). These correlations may restrict the use of related drugs when one drug becomes less susceptible. In our results, although the three antimicrobials presented similar MIC trends over time, no significant correlation was found except between vancomycin and daptomycin in group BB (*P* = 0.024) (Table 5). Interestingly, consistent with this result, most previously reported correlations amongst these drugs were found in bloodstream isolates (Shoji et al., 2015; San-Juan et al., 2016). Our data suggest that decreased vancomycin susceptibility probably restricts the usage of daptomycin in anti-MRSA therapy, but only for bloodstream-associated infections.

In conclusion, we found that MRSA infections are serious in the center and ST239-MRSA-III-t030-*agr*I clone is significantly dominated in SAB patients. In contrast with most other reports, although vancomycin and teicoplanin are frequently used in our center, the increasing susceptibility to the two glycopeptides and daptomycin over time was observed, suggesting that the application of glycopeptides does not necessarily lead to MIC creeps. SAB-MRSA isolates from different body sites presented different susceptibility profiles, indicating antimicrobials selection in anti-infection therapy should be considered comprehensively. MICs correlation was found only between vancomycin and daptomycin amongst bloodstream MRSA isolates, suggesting that the usage of daptomycin may be restricted in treating MRSA-associated bloodstream infections if decreased vancomycin susceptibility occurs.

## Author contributions

YP and YG conceived and designed this study. BJ, SY, and BY carried out the experiments, collected and analyzed the data. GH, ZiY, YZ, YC, and JC helped with the data interpretation. ZhY, XR, and XH drafted the manuscript. All authors have read and approved the final manuscript.

### Conflict of interest statement

The authors declare that the research was conducted in the absence of any commercial or financial relationships that could be construed as a potential conflict of interest.
